# Development of an informatics system for accelerating biomedical research.

**DOI:** 10.12688/f1000research.19161.2

**Published:** 2020-07-13

**Authors:** Vivek Navale, Michele Ji, Olga Vovk, Leonie Misquitta, Tsega Gebremichael, Alison Garcia, Yang Fann, Matthew McAuliffe

**Affiliations:** 1 Office of Intramural Research, Center for Information Technology, National Institutes of Health, USA, Bethesda, Maryland, 20892, USA; 2 General Dynamics Information Technology, Inc., Fairfax, Virginia, 22030, USA; 3 Sapient Government Services, Arlington, Virginia, 22201, USA; 4 Intramural IT and Bioinformatics Program, National Institute of Neurological Disorders and Stroke, National Institutes of Health, Bethesda, Maryland, 20892, USA

**Keywords:** Informatics system, Biomedical repository, Translational Research, FAIR

## Abstract

The Biomedical Research Informatics Computing System (BRICS) was developed to support multiple disease-focused research programs. Seven service modules are integrated together to provide a collaborative and extensible web-based environment. The modules—Data Dictionary, Account Management, Query Tool, Protocol and Form Research Management System, Meta Study, Data Repository and Globally Unique Identifier —facilitate the management of research protocols, to submit, process, curate, access and store clinical, imaging, and derived genomics data within the associated data repositories. Multiple instances of BRICS are deployed to support various biomedical research communities focused on accelerating discoveries for rare diseases, Traumatic Brain Injury, Parkinson’s Disease, inherited eye diseases and symptom science research. No Personally Identifiable Information is stored within the data repositories. Digital Object Identifiers are associated with the research studies. Reusability of biomedical data is enhanced by Common Data Elements (CDEs) which enable systematic collection, analysis and sharing of data. The use of CDEs with a service-oriented informatics architecture enabled the development of disease-specific repositories that support hypothesis-based biomedical research.

## Introduction

Biomedical informatics systems can be used for the management of heterogeneous data, testing of data analysis methods, dissemination of translational research, as well as for high-throughput hypothesis generation work ^1, 2^. In the past, many disease focused research programs have collected data in dissimilar ways, which has resulted in difficulties for data aggregation and comparative analyses. For example, non-standard methods of data collection in Traumatic Brain Injury (TBI) research have led to many different types of injuries to be classified within the same class of injury. To overcome this problem, in October 2007, the National Institute of Neurological Disorders and Stroke (NINDS), along with National Institute on Disability and Rehabilitation Research (NIDRR), the Defense and Veterans Brain Injury Center and the Brain Injury Association of America sponsored a workshop to examine barriers to TBI clinical trial effectiveness. The workshop recommendation for improving data discoverability and integration in TBI research resulted in the development and implementation of Common Data Elements (CDEs) and the Federal Interagency Traumatic Brain Injury Research (FITBIR) Informatics System ^3^.

A CDE is defined as a fixed representation of a variable collected within a specified clinical domain, interpretable unambiguously in human and machine-computable terms ^4^. It consists of a precisely defined question with a set of permissible values as responses. Typically, CDE development for biomedical disease programs involves multiple steps — identification of a need for a CDE or group of CDEs, bringing together stakeholders and expert groups for selection, various iterations and updates to initial CDE development with ongoing input from the broader community, culminating with final endorsement of the CDEs by the stakeholder community for its usage and widespread adoption ^5^. Use of CDEs enhances data quality and consistency, which facilitates data reuse for clinical and translational research.

CDEs are used in various programs of clinical research to include neuroscience ^6^, rare diseases research ^7^ and management of chronic conditions ^8^. For clinical data lifecycle management, the use of CDEs provides a structured data collection process, which enhances the likelihood for data to be pooled and combined for meta-analyses, modeling and post-hoc construction of synthetic cohorts for exploratory analyses ^9^. Investigators working to develop protocols for data collection can also consult the NIH Common Data Element Resource Portal for using established CDEs for disease programs ^10^.

In 2010, the Department of Defense and the NINDS initiated the development of the FITBIR. The goal was to develop a centralized repository for TBI research, in order to foster collaboration between researchers working in the field. Additionally, the design of FITBIR called for the use of CDEs during TBI data collection.

Prior to the development of FITBIR, the National Database for Autism Research (NDAR) system had demonstrated the use of CDEs for Autism research ^11^. Certain design features such as the use of a Global Unique Identifier (GUID) scheme were adopted from NDAR for FITBIR. However, the NDAR model was dedicated for access and submission to federated databases for Autism Research. FITBIR, on the other hand, required development of a multi-program centralized repository.

The Biomedical Research Informatics Computing System (BRICS) was designed to address the wide-ranging needs of several biomedical research programs. The overall concept was to develop services that could be integrated together and deployed as instances for individual research programs. FITBIR was the first initial BRICS instance and was leveraged to develop other instances (e.g., Parkinson’s disease program). The BRICS instance supports electronic data capture and use of data dictionaries for processing and storing data within disease- specific digital repositories.

Data Dictionaries (DD) comprise data elements, Form Structures (FS) and electronic forms (eForms). A data element has a name, precise definition and clear permissible values, if applicable. A data element directly relates to a question on a paper, eForm, and/or field(s) in a database record. FS serve as the containers for data elements, and the eForms are developed using FS as their foundation. The data dictionary provides defined CDEs, as well as Unique Data Elements (UDEs), for specific BRICS instance implementation. Reuse of CDEs is significantly encouraged, and in the case of FITBIR’s data dictionary, it incorporates and extends the CDE definitions developed by the National Institute of Neurological Disorders and Stroke (NINDS) CDE Project ^6^.

This paper discusses the overall system design and an architecture that supports the various BRICS instances. The functionalities developed to use the CDEs for electronic data submission, processing, validation and storage within designated repositories have been presented. System access is highlighted for searching across research studies within a BRICS instance. An example has been provided for BRICS implementation within a disease area (Parkinson’s disease) research. Also shown is the role of individual system components that enable data to be findable, accessible, interoperable and reusable.

### BRICS System Design and Architecture -

The system design was predicated on the adoption of a CDE-based data collection method. To satisfy this requirement, an electronic data collection tool (ProFoRMS) was developed to interface with DD, which enabled deployment of multiple instances of the system to disease area programs. This method of using CDEs early in the data life cycle facilitated data harmonization and minimized the need for elaborate post processing and curation work. Services were developed to support the various stages in the data life cycle. De-identification of each patient within a research study is supported by the use of a Global Unique Identifier (GUID). A de-identification tool was developed for researchers to use prior to submission of data to a specific BRICS instance. No personally identifiable information could be retained in the BRICS repositories.

**Figure 1.**
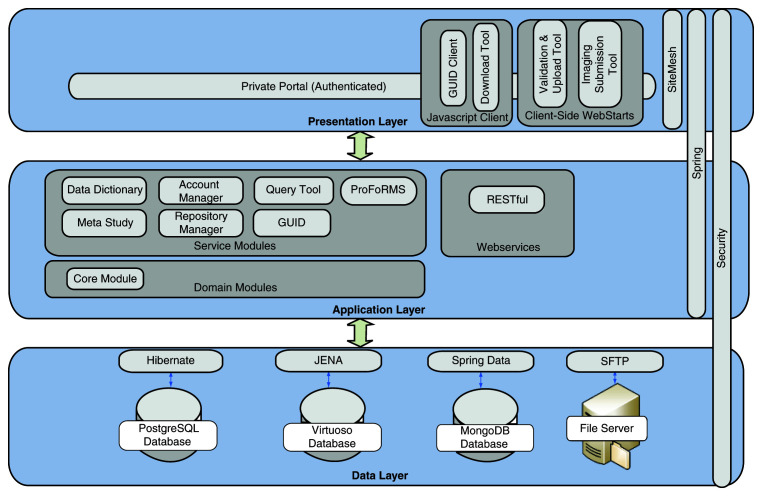
A schematic representation of the informatics system architecture.

Since BRICS development started in 2011, the Java Web Start technology was used for deploying the tools shown in the presentation layer of the architecture ( Figure 1). Although in subsequent editions of Java to Oracle Java SE 8, Java Web Start was deprecated, free public updates and auto updates to the Java SE 8 are provided by Oracle Inc., until at least the end of December 2020. GUID and Download tools that initially used the Web Start technology have been migrated to the Javascript client. The Submission tool will also be migrated to Javascript client by end of 2020. During the transition period, users continue to maintain the Oracle Java SE 8 installed on their local computers.

An open-source database, PostgreSQL was preferred over Oracle database during BRICS development, primarily to minimize individual licensing costs when deploying instances of the system to various biomedical programs. However, three separate PostgreSQL databases were used, one for DD, and the other two for ProFoRMS, Data Repository (DR) and Meta Study functionalities, respectively. Separate databases were needed because the data dictionary is shared and ProFoRMS was developed as an application that was integrated with the system.

The Virtuoso database uses Resource Description Framework (RDF) for accessing data that comes from a DR, DD, and Meta-data modules. Virtuoso contains data that are linked together in RDF, to support the query tool. The repository data is linked to meta-data (studies and datasets) and data dictionary, which is processed and stored in Virtuoso for querying. An advantage of using the RDF triple model is its flexibility to adapt to user-driven data requirement changes that can be made in the study repository or Query Tool. Once the data is added to the RDF graph as triples, regardless of where the data is stored, it can easily be retrieved and processed by the Query Tool.

Since the initial release of the BRICS platform, we have initiated a migration to the MongoDB database to take advantage of schema-free development. Currently, the GUID module has been migrated to use MongoDB. Other BRICS functionalities will be migrated to use MongoDB thereby eliminating the need for using PostgreSQL database in the BRICS architecture.

An overview of the current informatics system architecture is provided in Figure 1. The architecture is defined by the three layers—(a) Presentation Layer, (b) Application Layer and (c) Data Layer. The Presentation Layer provides a secure entry point through the BRICS portal. A login page is used to enter valid credentials with a Central Authentication System (CAS) to support single sign-on for users to access all the BRICS modules. A role-based access has also been implemented by using Spring Security (a Java/Java enterprise edition framework that provides authentication and authorization features) throughout the system to provide additional level of controlled access to each of the modules. The Global Unique Identifier (GUID) client, Validation/Upload, Download and Image Submission tools are accessible via the BRICS portal.

The Image Submission Package Creation Tool, a plugin to the Medical Image Processing Analysis and Visualization (MIPAV) application ^12, 13^, leverages medical image file readers found in the MIPAV software application (v 8.0.2) to support the semi-automated submission of image data into the DR. The plugin supports more than 35 file formats commonly used in medical imaging, including DICOM, NIfTI, Analyze, AFNI and more. The Image Submission Tool extracts available image header metadata from the image and attempts to map that metadata onto the CDEs in the selected imaging Form Structure. The quality and amount of image header metadata that can be extracted out of an image volume will depend on the medical image file format, the scanner on which the images were acquired and the de-identification process performed.

The Application Layer is responsible for the logic that determines the capabilities of the BRICS modules and tools. Seven service modules within the Application Layer are integrated together to provide a collaborative and extensible web-based environment. These modules are the DD, Account Management, Query Tool, Protocol and Form Research Management System (ProFoRMS), Meta Study, DR and GUID. To communicate and exchange information between the modules, representational state transfer (RESTful) interface for the Web services is used ^14^. Additional information about the various service modules is available from the BRICS site.

The Data Layer consists of open-source databases including PostgreSQL, Virtuoso and MongoDB. Since a typical query use case requires data from a repository, DD and Meta-Study module, it is much more efficient to store and access data in a single Virtuoso database. Instead of using resource intensive joins in the PostgreSQL, data can be accessed in Virtuoso by traversing RDF graph database. Having related data linked together in one place allows Query Tool to quickly query repository data that would otherwise be slow. RDF is also used to support searching of studies, form structures and data elements.

Also utilized are open-source libraries such as Hibernate and Apache Jena for storing and retrieving data from databases. Hibernate is an object-relational mapping framework used to map PostgreSQL data into Java objects. Using Hibernate reduces the amount of software code that would otherwise be required to translate tabular data from SQL into Java objects. Jena is a Java framework that enables interaction with semantic web applications; it is the Hibernate equivalent for semantic web, mapping the Virtuoso data into Java objects. Both of these frameworks support users’ requests for retrieving and storing data. A single library was not available to support data persistence therefore Hibernate was used for the PostgreSQL, and JENA was used to support Virtuoso’s RDF structure.

The data layer is supported by the physical infrastructure located within the National Institutes of Health (NIH). It is certified to operate at the Federal Information Security Modernization Act (FISMA) moderate level ^15– 17^. In accordance with FISMA moderate systems, the BRICS system adheres to the NIST 800-53 security standards and guidelines. The BRICS system is certified for the Title 21 Code of Federal Regulations (21 CFR Part 11) and as part of the CFR requirements, a stringent audit trail has been implemented within the BRICS system to verify that digital objects have not been altered or corrupted.

Researchers can use the GUID tool (shown as a client in Figure 1) to support the de-identification of data and assign a unique identifier for each study participant. The GUID is a random alphanumeric unique subject identifier that is not directly generated from personally identifiable information (PII) ^18^. Generating a GUID involves inputting a required set of reproducible and invariant subject information, typically found on the subject’s birth certificate, into a client application. The PII fields include complete legal given (first) name of subject at birth, middle name (if available), complete legal family (last) name of subject at birth, day of birth, month of birth, year of birth, name of city/municipality in which the subject was born and country of birth. The PII data is not sent to the GUID server but rather one-way encrypted hash codes are created and sent from the GUID client to the server (represented as a service module, Figure 1), allowing the PII to reside only on the researcher’s site. A random number for each research participant is generated by the server and is returned to the researcher. The same GUID is provided if the participant is enrolled in multiple studies. The GUID server can be configured to support multi-center clinical trials and investigations that enroll research participants across various programs.

### Data Submission and Processing

Institutional grants (e.g. from the DOD or NIH) that support disease-specific research require data submission to a specific BRICS instance. For example, TBI researchers receiving grants from the DOD and NIH are required to submit data to FITBIR. A concerted approach of submitting study data to a BRICS instance facilitates data reuse, validation and aggregation with other studies, thereby supporting meta-analysis of clinical studies. Currently, BRICS instance repositories contain patient assessment (form) data, imaging, electroencephalogram (EEG), magnetoencephalography (MEG) and derived genomics data. Researchers are responsible for data submission activities, which includes FS approval, eForms review, curation, mapping of data elements and providing associated study documentation that describes data collected in the study. However, review and approval for using an FS is carried out by the data curator and the disease area program lead.

**Figure 2.**
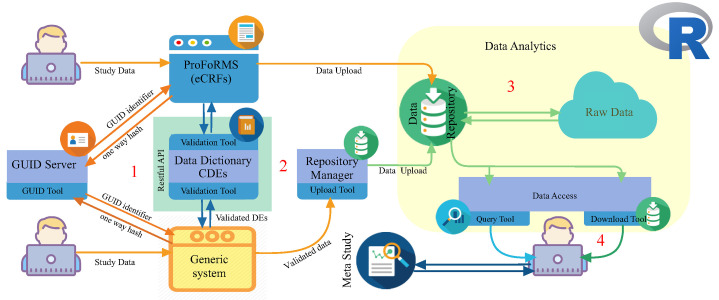
Schematic representation of 1. Submission Information Package (SIP), 2 - Archival Information Package (AIP) preparation, 3 - storage of AIPs and 4 - Dissemination Information Packages (DIP) access.

For clinical research work, the ProFoRMS tool can be used for scheduling subject visits, collecting data, adding new data, modifying previously collected data entries and correcting discrepancies ( Figure 2, stage 1). Using ProFoRMS provides for automatic validation with data dictionaries associated with each of the BRICS instance(s). The data dictionaries were developed by collaborative efforts of disease area experts, including the NINDS, DOD and National Library of Medicine ^6, 10^.

Researchers have the option to collect data by generic system (e.g. REDCap); however, the output file from the generic system will have to be validated with the specific BRICS instance data dictionaries before being uploaded to data repository ( Figure 2, stage 2).

The data submission file format is comma-separated values (CSV) and structured so that the data is consistent with CDE-variable names and data values. The Validation Tool supports the data repository and ProFoRMs modules, by validating data against CDEs which have defined ranges or permissible values. If the data contains errors, the user must correct the errors before a submission package can be generated and the data be submitted. This validation, as part of the data submission process, is a major step towards making data reusable.

Once the data has been validated, it is uploaded via the submission upload tool. An original copy of the user submitted data (raw data) is maintained in the repository. Nightly, the raw data is loading into the Query Tool’s database ( Figure 2, stage 3). Study-specific clinical, imaging and derived genomics data are available for search and retrieval.

User support is provided for data stewardship activities that include training and assistance to authorized users for CDE implementation, data validation and submission to the repositories. Access is controlled by a Data Access Committee (DAC) that reviews user applications to a specified BRICS instance (defined by the biomedical program). In addition, access to the system is role-based and specific permissions are associated with roles such as PI, data manager and data submitter.

During packaging of data in the CSV file, GUIDs are assigned to research subjects (patients), using the GUID client with the users responsible for storing PII data locally within their institutional systems. Data curation is carried out by identifying the available standard forms and CDEs in the Data Dictionary. In the event no corresponding CDEs are available, then the user can define unique data elements and obtain approval during the submission process.

The Data Repository module serves as a central hub, providing functionality for defining and managing study information and storing the research data associated with each study ( Figure 2, stage 3). Authorized investigators can submit data to a BRICS instance and organize one or many datasets into a single entity called a Study. In general terms, a Study is a container for the data to be submitted, allowing an investigator to describe, in detail, any data collected, and the methods used to collect the data, which makes data accessible to users. By using the repository user interface, researchers can generate digital object identifiers (DOI’s) for a study, which can be referenced in research articles. Since the NIH has a DataCite membership, users can directly use the repository interface for producing the DOI’s.

Study metadata is entered within a BRICS instance manually through graphical user interfaces. The metadata fields include title, organization, PI, data, funding source and ID’s, study type(s) and keywords that enable users to search for detailed information (e.g. clinical trial Grant ID(s), start and end dates for grants, therapeutic agents, sample size, publications and/or forms used).

Each of the BRICS instances exposes metadata and summary consistent with their respective program goals. For example, FITBIR provides a metadata visualization tool that graphically supports searching study identification (shown here https://fitbir.nih.gov/visualization).

Depending on the BRICS instance, investigators can download summary statistics for specific studies. BRICS-based repositories (e.g. eyeGene) host high throughput gene expression, RNA-Seq, SNPs and sequence variation data sets ( Figure 2, stage 3).

### Data Sharing and Access

By default, a BRICS instance assigns the sharing preference as ‘private,’ where only users to that specific study can access the data. When the data is in the private state, the PI has the option to share data with specific collaborators (preferential sharing). Each instance supports data sharing policies consistent with its program policies. Researcher data is maintained in a private state until a year after the research grant end date. Subsequently, it is moved to the shared state whereby the Data Access Committee (DAC) authorizes access to other users of the data. The DAC is typically comprised of government program officials responsible for each of the BRICS instances, who evaluate the data access requests and approve or disapprove them. Detailed information on the BRICS instances can be gleaned from individual program websites for the biomedical program areas that is provided in a latter section of the paper.

Raw data is available for querying within 24 hours of data submission. For the data to be available via the Query Tool module, the raw data is processed through the NextGen Connect tool (integrated interface engine) and Resource Description Framework (RDF) data interchange tool ( Figure 2, stage 4). Shared data is available to all system users (approved by DAC) to search, filter and download via the Query Tool (QT) functionality.

The QT offers three types of functionalities—(a) querying and filtering data, (b) data package downloads based on query and (c) data package to the Meta Study module.

The QT enables users to browse studies, forms and CDEs, to select clinical data, use filters, and to sort and combine records. Using the GUID and a standard vocabulary via CDEs in forms, the QT provides an efficient means to reuse data by searching through volumes of aggregated research data across studies, finding the right datasets to download and performing offline analysis using additional tools (e.g. SAS, SPSS, etc.).

There are several ways to search for data using the QT. Figure 3a is an example of a BRICS instance for the Parkinson’s disease program. Users can use the QT to search for a specific study, across studies by using a form or individual data element.

**Figure 3a.**
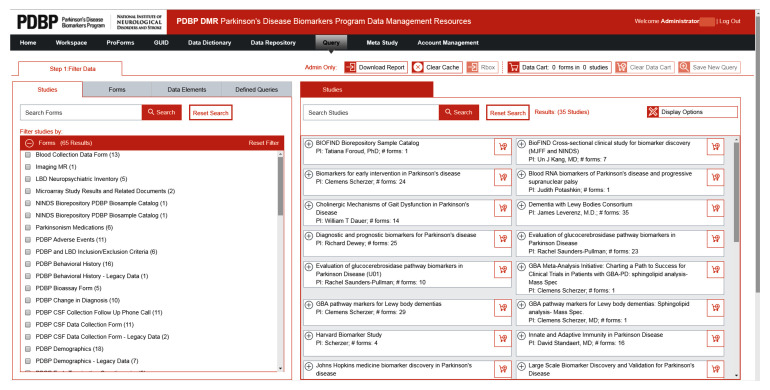
The Query Tool functionality is used to browse studies and forms, search data within forms and across studies. Example above is from the Parkinson’s Disease Biomarker Program BRICS instance.

**Figure 3b.**
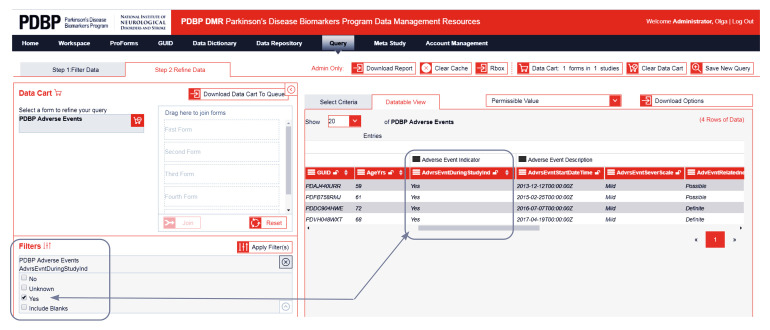
The Query Tool can be utilized by users to select from a list of data elements that exist or are part of a form structure.

Each column of data in a QT result represents a well-defined data element in the Data Dictionary. Users can refine results by selecting from the list of allowed element-permissible values, like male or female, or move sliders to select a range of numeric values, like age or outcome scores (shown in Figure 3b).

In addition to providing tools to aid data discovery, the QT supports interactive features that facilitate analysis and practical use of the data through attribute-based filtering capabilities, based on the data element type.

BRICS supports domain-(disease-)specific repositories that host various datasets (e.g. clinical, cognitive, demographic). Data can be shared in a CSV file format for download, and/or stored in the Meta Study module for further analysis, research, and reference.

The primary purpose of Meta Study module is to provide a virtual workspace where research data and metadata acquired across studies can be stored and findings can be associated with a DOI and cited for journal publications. A Meta Study contains findings from studies that can be aggregated by researchers to conduct additional analysis.

### Implementation example

The Parkinson’s Disease Biomarker Program (PDBP) was developed to accelerate the discovery of promising new diagnostic and progression biomarkers. This requires data replication and validation prior to clinical trial use ^19^. The system consists of two major components—(a) a Drupal-based portal and (b) the PDBP Data Management Resource (DMR). The portal is publicly accessible to varied users such as stakeholders, participants, and researchers to obtain information about policy, summary data and news (see PDBP site). The PDBP DMR is a BRICS instance and is comprised of the modules (shown in Figure 2), and incorporates the Parkinson’s disease CDEs into its Data Dictionary ^20^. The CDEs are easily accessible from multiple open resources— the PDBP Data Dictionary ^20^, the NINDS CDE project ^6^ and the NIH CDE repository ^10^. The DMR is securely managed with capabilities for account verification, GUID generation, data submission, validation, workflows, access and biospecimen data management. A GUID is generated for each subject on their initial visit and is attached to the de-identified data. The GUID makes data reusable by enabling the aggregation of all research data (clinical, imaging, genomic and biomarker) for a specific subject, both within a single study and across many PDBP studies.

The ProFoRMS module (shown in Figure 2) is used to schedule Parkinson’s Disease program subject visits and capture data (including the GUID) via a web-based assessment e-form tool. It provides capabilities for real-time data entry and automatic data harmonization via CDEs, and ensures data quality prior to storage within the PDBP repository. Each of the questions in the PDBP DMR assessment form is associated with a CDE that supports reusability and interoperability of PDBP data ^19, 21^. ProFoRMS also provides automatic assignment of specific forms to individualized cohorts based on protocol design and quality assessment of data prior to uploading to the PDBP Data Repository.

The authorized PDBP users can use the QT for accessing data across studies and aggregate data based on assessment forms and CDEs, allowing for the linkage of biosample data to demographics data. More complex queries can be created by linking clinical data from ProFoRMS with imaging data, and with corresponding biospecimens/biosamples.

Data can be downloaded directly from the PDBP data repository and/or from the QT to be analyzed by researchers using their preferred tools. Because the DMR database contains only de-identified data, all data uploaded to the DMR can be shared with the scientific community. Use of standard operating procedures has resulted in harmonization of biospecimens/biosamples with the DMR Biosample Order Manager Tool, which enables linking clinical and biorepository data ^22, 23^. The PDBP data, queries and other metadata described for the research can be loaded into the Meta Study module. Through the Meta Study user interface, researchers can generate DOIs that can be referenced in research articles.

### BRICS instances

A brief description of resulting data repositories by the implementation of BRICS instances is provided below.

***Federal Interagency Traumatic Brain Injury Research (FITBIR).*** is a BRICS instance developed to advance comparative effectiveness research in support of improved diagnosis and treatment for those who have sustained a TBI ^24^. The FITBIR repository stores data provided by TBI researchers and has accepted high-quality research data from several studies, regardless of funding source and location. The DoD and NINDS support TBI human subject studies (both retrospective and prospective) and have required the research grantees to upload their clinical, imaging and genomic data to FITBIR. As of 2020, there were 116 studies in FITBIR, with data contribution from over hundred PIs. Data on 80,550 subjects, including more than 113,842 clinical image 3D data sets are part of the repository. There are a total of 3,525,427 records in FITBIR. Data provided to FITBIR for broad research access are expected to be made available to all users within six months after the award period ends. Updated information about records is available at the FITBIR site: https://fitbir.nih.gov/. The site also provides information on TBI-specific data sharing policies.

***The Parkinson’s Disease Biomarkers Program Data Management Resource (PDBP DMR).*** is a BRICS instance that is supported by NINDS, and is a resource for promoting Parkinson's disease biomarker discovery efforts. At the center of the PDBP effort is its DMR. The PDBP DMR uses a system of standardized data elements and definitions, which makes it easy for researchers to compare data to previous studies, access images and other information and order biosamples for their own research. PDBP’s needs have accelerated BRICS system development, such as enhancements to the ProFoRMS data capture module and an investment in a plug-in for managing biosamples. The PDBP DMR now contains 49 studies comprising 14,377 subjects, 65 imaging data sets and 34,206 biorepository samples. Also, PDBP currently has a total of 1,368,906 records that are updated periodically at the following site: https://pdbp.ninds.nih.gov/.

***eyeGENE.*** is a BRICS instance for supporting the National Ophthalmic Disease Genotyping and Phenotyping Network ^25^. It is a research venture created by the National Eye Institute (NEI) to advance studies of eye diseases and their genetic causes by giving researchers access to DNA samples and clinical information. Data stored in eyeGENE is mapped to Logical Observation Identifiers Names and Codes terminology (LOINC) interoperability data standards ^26^. Currently, eyeGene has 11,236,576 records with 6,416 enrolled subjects. Program specific information on data sharing is available here: https://eyegene.nih.gov.

***The Informatics Core of Center for Neuroscience and Regenerative Medicine (CNRM).*** has a BRICS instance to support the CNRM medical research program with collaboration between the DOD, NIH and Walter Reed National Military Medical Center. The Informatics Core provides services such as electronic data capture and reporting for clinical protocols, participation in national TBI research and a data repository community, integration of CNRM technology requirements and maintenance of a CNRM central data repository ^27^. Additional information is at the site, https://cnrm-dr.nih.gov/. In addition, the Informatics Core has played an important role in the development of multiple BRICS modules used by FITBIR.

***The Common Data Repository for Nursing Science (cdRNS).*** BRICS instance supports the National Institute of Nursing Research (NINR) mission—to promote and improve the health of individuals, families, and communities ^28^. To achieve this mission, NINR supports and conducts clinical and basic research and research training on health and illness. This research spans and integrates the behavioral and biological sciences to further the development of scientific basis for clinical practice ^29^. The NINR is a leading supporter of clinical studies in symptom science and self-management research. To harmonize data collected from clinical studies, NINR is spearheading an effort to develop CDEs in nursing science. Currently, there are 846 subjects with 11,504 records in the cdRNS instance of BRICS. Additional information is available at: https://cdrns.nih.gov/.

***The Rare Diseases Registry Program (RaDaR).*** has a BRICS instance supporting the National Center for Advancing Translational Sciences (NCATS). It is designed to advance research for rare diseases ^30^. Because many rare diseases share biological pathways, analyses across diseases can speed the development of new therapeutics. The goal is to build a web-based resource that integrates, secures and stores de-identified patient information from many different registries for rare diseases. Currently there are 25,354 subjects enrolled in the registry. The RaDaR program uses the BRICS GUID functionality, a complimentary software provided by the NCATS that enables registry owners to download and generate the GUID. Registry owners can access the GUID software at https://rarediseases.info.nih.gov/radar/global-unique-identifier-generator.

***National Trauma Research Repository.*** (NTRR) has a BRICS instance deployed to support National Trauma Institute (NTI) ^31^. The NTRR deployment is within a secure Amazon Web Services (AWS) Cloud, that provides Infrastructure as a Service (IaaS) for processing, storage and computing needs ^32^.

## Discussion

Software tools for collecting and managing project-related clinical research data are also provided by tranSMART platform, which utilizes an ontology-based mapping to an institution specific or industry standard formats ^33^. However, for disease-focused research using CDEs during data collection eliminates the need for ontology-based mapping. Users can use REDCap software tool ^34, 35^ to collect data and submit to a BRICS instance, which can be validated with the DD provided for the individual instances.

For long-term preservation of data, the Open Archival Information System (OAIS) model highlights the importance of six functions—ingest, access, data management, archival storage, administration and preservation planning ^36^. Specifically, the model provides a framework for preserving information for a designated community (group of potential consumers and multiple stakeholders). The model is unique because it is content and technology agnostic. We have applied the OAIS model for long term preservation of biomedical data collected for disease area research, implementing the concept of Submission, Archival and Dissemination Information Packages (SIP, AIP, DIP) for processing data for designated biomedical communities ^37^. Figure 2 illustrates the process of clinical data SIPs and AIPs, produced for each of the instances by using eCRFs. Imaging data SIPs are produced by the Image Submission tool. The CDEs and data dictionaries for the various BRICS instances support the development of Archival Information Packages (AIPs), which are preserved in distinct data repositories identified by the biomedical research programs ^38^.

### Supporting the FAIR principles

The FAIR (Findable, Accessible, Interoperable and Reusable) principles state that stewardship of digital data should promote discoverability and reuse of digital objects, which includes data, metadata, software and workflows ^39^. In addition, the principles posit that data and metadata should be accompanied by persistent identifiers (PIDs)—indexed in a searchable resource retrievable by their identifiers, and which use vocabularies that meet domain relevant community standards. The principles should be considered during development of informatics systems to further promote data discovery and reuse. In Table 1, we have correlated the various BRICS functional components to the FAIR principles to illustrate the extent to which each of the components contribute towards the principles.

**Table 1.** Informatics functional components that support the FAIR (Findable, Accessible, Interoperable and Reusable) principles. The FAIR principles listed in the table are from the cited reference ^39^.

	Functional Components					
FAIR Principles	GUID	Data Dictionary	Data Repository	ProFoRMs	Query Tool	MetaStudy
**Findable**						
Data are assigned a globally unique and eternally persistent identifier	x	x	x			x
Data are described with rich metadata		x	x	X	x	x
Metadata clearly and explicitly include the identifier of the data it describes			x			x
Metadata are registered or indexed in a searchable resource		x	x			
**Accessible**						
Metadata are retrievable by their identifier using a standardized communications protocol		x				
The protocol is open, free and universally implementable		x				
The protocol allows for an authentication and authorization procedure, where necessary	x	x	x	X	x	x
Metadata are accessible, even when the data are no longer available		x				
Interoperable						
Metadata use a formal, accessible, shared and broadly applicable language for knowledge representation		x	x			
Metadata use vocabularies that follow FAIR principles		x	x	X		
Metadata include qualified references to other metadata		x			x	
Reusable						
Metadata have a plurality of accurate and relevant attributes		x	x		x	x
Metadata are released with a clear and accessible data usage license		x	x			x
Metadata are associated with their provenance		x	x			x
Metadata meet domain-relevant community standards		x	x			

For example, the use of Data Dictionary supports many of the FAIR principles. Access to data and metadata requires unique identification that is human and machine-readable ^40^. In the context of the BRICS, GUID does not imply findability on the web and therefore cannot be considered globally unique as implied by the principles. However, the system supports findability of research participant data within a BRICS instance. Authorized researchers can use GUID to link together all submitted information for a single participant, even if data was collected at different locations and/or for different purpose(s).

While GUIDs as defined here are locally unique within BRICS, DOIs are globally unique.

The DOIs generated by BRICS are through the Interagency Data ID Service (IAD), which is operated by the U.S. Department of Energy Office of Scientific and Technical Information (OSTI). The IAD service acts as a bridge to DataCite, which is one of the major registries of DOIs. The DOIs are assigned to individual research studies and are findable within the established repositories, available also from open sites with core metadata supported via Data Tag Suite (DATS) 2.2 ^41^.

Data quality and consistency of submissions is enhanced by validation with domain specific DD. BRICS also provides for an automated means of mapping CDEs to other informatic systems data dictionaries, e.g. Clinical Data Interchange Standards Consortium (CDISC) ^42^. As indicated earlier that CDEs are available through public websites (e.g. National Library of Medicine (NLM), NINDS CDE project, CDISC, etc.), to make data interoperable and reusable.

## Conclusion

Data confidentiality, integrity and accessibility are essential elements of responsible biomedical research data management. Community-wide data sharing requires development and application of informatics systems that promote collaboration and sustain data integrity of research studies within a secure environment. The BRICS informatics system enables researchers to efficiently collect, validate, harmonize and analyze research datasets for various biomedical programs. Integration of the CDE methodology with the informatics design results in sustainable digital biomedical repositories that ensure higher data quality. Aggregating data across projects, regardless of location and data collection time can define study populations of choice for exploring new hypotheses based-research.

## Data availability

### Underlying data

All data underlying the results are available as part of the article and no additional source data are required.

### Software availability

Source code available from: https://github.com/brics-dev/brics

Archived source code at time of publication: http://www.doi.org/10.5281/zenodo.3887046 ^43^

License: Other (open). Full license agreement is available from GitHub https://github.com/brics-dev/brics/blob/master/license.txt
