# Supplementary material for: Development of an informatics system for accelerating biomedical research
Source: F1000Res. 2020 Jul 13;8:1430. Originally published 2019 Aug 14. [Version 2] doi: 10.12688/f1000research.19161.2 (PMC7376384; doi:10.12688/f1000research.19161.2)
Supplement: Peer review history

# Peer review: Reviewer response for version 2

**DOI:** 10.5256/f1000research.27924.r66956

**Published:** 2020-07-22

**Authors:** Hyeoneui Kim

**Affiliations:** 1 School of Nursing, Duke University, Durham, NC, USA

The authors successfully addressed the questions and the suggested changes in this revised version. Again, this is a vital work that showcases implementing an infrastructure that supports the FAIR principle of biomedical data. The details provided in this work will inspire many similar efforts in other biomedical domains.

Is the rationale for developing the new method (or application) clearly explained?

Yes

Is the description of the method technically sound?

Yes

Are the conclusions about the method and its performance adequately supported by the findings presented in the article?

Yes

If any results are presented, are all the source data underlying the results available to ensure full reproducibility?

Partly

Are sufficient details provided to allow replication of the method development and its use by others?

Yes

Reviewer Expertise:

biomedical informatics, standardized data representation

I confirm that I have read this submission and believe that I have an appropriate level of expertise to confirm that it is of an acceptable scientific standard.

# Peer review: Reviewer response for version 2

**DOI:** 10.5256/f1000research.27924.r66955

**Published:** 2020-07-13

**Authors:** Timothy W. Clark

**Affiliations:** 1 School of Medicine and School of Data Science, University of Virginia, Charlottesville, VA, USA

This extensive and thorough revision to the original version addresses all the points I made in my earlier review.

Is the rationale for developing the new method (or application) clearly explained?

No

Is the description of the method technically sound?

Partly

Are the conclusions about the method and its performance adequately supported by the findings presented in the article?

No

If any results are presented, are all the source data underlying the results available to ensure full reproducibility?

No source data required

Are sufficient details provided to allow replication of the method development and its use by others?

No

Reviewer Expertise:

Biomedical informatics. Semantic technologies. Cloud computing frameworks. Neuroscience. Data science.

I confirm that I have read this submission and believe that I have an appropriate level of expertise to confirm that it is of an acceptable scientific standard.

# Peer review: Reviewer response for version 1

**DOI:** 10.5256/f1000research.20997.r55222

**Published:** 2019-12-03

**Authors:** Timothy W. Clark

**Affiliations:** 1 School of Medicine and School of Data Science, University of Virginia, Charlottesville, VA, USA

This is an article which one eventually learns is about the BRICS system, developed for NINDS by software consultants at General Dynamics and Sapient. It is poorly organized, and seems to take little care to actually explain the system's motivation and use in a coherent fashion understandable to the reader. 

 The authors begin with an overgeneralized and basically non-informative abstract and introduction which make a number of broad statements about translational research and its requirements for enabling informatics. But one does not discover the actual real focused topic of the article until far down in the text.

 One does not learn, for example, until page 8, the specific motivation of the system:

 "The initial deployment of BRICS was to support the U.S. Department of Defense’s (DoD) and the National Institute of Neurological Disorders and Strokes (NINDS), FITBIR project. The core functionalities for FITBIR were reusable for developing PDBP, as well other biomedical programs."  

 Obviously there has been a major software development effort conducted by contractors funded and managed by NINDS, and this article is attempting to report on what was accomplished. The implementation is not interesting in itself, using mostly 20-year-old technology. However, the scale of the software effort and its importance to researchers working on certain NIH funded projects, would seem to require something far better as a report out, whether or not it was accepted by a peer-reviewed journal. 

 Crucial rationales for technical decisions made, and contexts in which the system is used, are omitted or poorly motivated, or described inaccurately. For example, we learn on page 4 that:

 "The Data Layer consists of open source databases such as PostgreSQL, Virtuoso databases, file servers, and data persistence frameworks."

 Virtuoso is not an open-source database. It is an enterprise-class RDF graph store. Nowhere up to now has the need for an RDF graph store been explained, and it is not touched on again until page 6, where we learn that:

 "The raw data is processed through the ‘NextGen Connect’ tool (integrated interface engine) and Resource Description Framework (RDF) data interchange tool." 

 And that is all we ever learn about the use of RDF or any related semantic technologies in this system. Are there OWL Ontologies involved? Why was the decision made to use them and which ones were selected? Why is Virtuoso used in conjunction with PostgreSQL relational store? What makes this combination necessary? We hear nothing of this. 

 This reviewer prepared a line-by-line discussion of the text which can be found here. Suffice it to say that there are many significant issues with this article - issues of poor exposition, lack of required detail or context, imprecision, or simply misleading statements.

 Another example:

 "The Meta Study module is used for meta-analysis of the data as well as a collaboration tool between scientific groups. "  

 That sentence is all we ever hear of the ability to perform meta-analysis, or any of the challenges it poses. 

 Or this:

 "Deploying in the cloud environment enhances data access, sharing, and reuse of biomedical research data at larger scale". 

 In fact the reason to deploy things in the cloud is for rapid horizontal and vertical scaling. lt has nothing to do with data sharing and reuse. The authors cite an article by one of them (Navale & Bourne 2018), to back up their incorrect claim - but that article directly contradicts this claim. 

 I strongly recommend to the authors that they engage a high-quality technical writing firm competent in bioinformatics to revise the text, paying close attention to precision, providing needed context for technical choices, context of usage and overall motivation of the system, and in general, thoughtful informative exposition.

Is the rationale for developing the new method (or application) clearly explained?

No

Is the description of the method technically sound?

Partly

Are the conclusions about the method and its performance adequately supported by the findings presented in the article?

No

If any results are presented, are all the source data underlying the results available to ensure full reproducibility?

No source data required

Are sufficient details provided to allow replication of the method development and its use by others?

No

Reviewer Expertise:

Biomedical informatics. Semantic technologies. Cloud computing frameworks. Neuroscience. Data science.

I confirm that I have read this submission and believe that I have an appropriate level of expertise to state that I do not consider it to be of an acceptable scientific standard, for reasons outlined above.

# Response

**Published:** 2020-07-07

**Authors:** Vivek Navale

**Affiliations:** National Institutes of Health, USA

1. Thank you for the comments. We have revised the manuscript extensively and addressed the detailed comments that you provided us. The organization of the manuscript has been made easier for readers to follow, appropriate headings have been contextualized throughout the paper. The introduction has been refocused, motivation highlighted and the specific points that were brought to our attention have been addressed in the manuscript.

2. We have revised the abstract and focused on the BRICS functional components, services deployed for research data life cycle management and demonstrated the application to various biomedical research programs. The common data element concept has been contextualized and the significance to this work has been highlighted.

3. Thank you for the comments on the BRICS Architecture section. We have made major revisions to this section to explain the design choices during the development work, lessons learned and accurately depicted the current architecture in Figure 1.

4. We have clarified in the Data Submission section that institutional grants (e.g. DOD, NIH) support disease specific research and mandate data to be submitted to a specific BRICS instance. 

5. We have revised the section to state that after data has been validated and uploaded to the repository, an original copy of the user submitted data (raw data) is maintained in the repository that can be accessed by the Query Tool.

6. Thank you for your suggestions. We have revised the section to clarify that data quality and consistency of submissions is enhanced by validation, using domain specific Data Dictionaries.

7. We agree that the biocaddie.org is not available, the project has been discontinued, hence access to BRICS repositories via bioCADDIE will not be possible. Therefore, we have removed the statements from the revised manuscript.

8. We have revised the section on BRICS instances and specified that the National Trauma Research Repository utilizes Cloud Computing for providing infrastructure as-a-service.

# Peer review: Reviewer response for version 1

**DOI:** 10.5256/f1000research.20997.r56129

**Published:** 2019-11-18

**Authors:** Hyeoneui Kim

**Affiliations:** 1 School of Nursing, Duke University, Durham, NC, USA

This paper introduces the Biomedical Research Informatics Computing System (BRICS), a comprehensive platform that supports researchers collect, store, analyze, and securely share research data. The underlying principle that motivated and enabled the implementation of this system is the FAIR (Findable, Accessible, Interoperable, and Reusable) principle for biomedical data.

 The authors provided clear and highly informative descriptions of the architecture and the approaches to implementing the key functional components. The related initiatives and programs that aim at improving data use and reuse and the gaps found in them provide a convincing context for BRICS development. The figures presented in the paper adequately describe the structure, functions, and workflows of BRICS. The research programs and initiatives that already utilize BRICS introduced in the paper are strong evidence that supports the BRICS approach. All in all, this is a well-written, very informative paper. The changes suggested below could help strengthen this paper even more:

  

1. The conventional section structure that includes methods and results might not fit well with this paper. If F1000Research allows some flexibility in the manuscript structure, it will help readers follow the progress of the content by changing the Method section to something like BRICS Functionalities and Components (or something along this line) and Result to BRICS Instance (or use cases). Also, the query module explained in the result section can be included in the BRICS functionalities and components section. 
2. A brief description of how the study level metadata (e.g., sample size, study design, study location, etc.) are captured would be helpful as study-level metadata are among the most frequently used parameters for data/dataset search.
3. Accessing a broader scope of data is one of the main motivations that researchers would adopt this type of data platform. Therefore, although it is not related to the technological development of BRICS, introducing more information on the data sharing policies (i.e., data use agreement and DAC's responsibilities) would be beneficial.
     
4. Please correct minor errors, such as typos and introducing BRICS first without fully spelling out the acronym.

Is the rationale for developing the new method (or application) clearly explained?

Yes

Is the description of the method technically sound?

Yes

Are the conclusions about the method and its performance adequately supported by the findings presented in the article?

Yes

If any results are presented, are all the source data underlying the results available to ensure full reproducibility?

Partly

Are sufficient details provided to allow replication of the method development and its use by others?

Yes

Reviewer Expertise:

biomedical informatics, standardized data representation

I confirm that I have read this submission and believe that I have an appropriate level of expertise to confirm that it is of an acceptable scientific standard, however I have significant reservations, as outlined above.

# Response

**Published:** 2020-07-07

**Authors:** Vivek Navale

**Affiliations:** National Institutes of Health, USA

1. Thank you for the suggestions. To improve clarity, we have redefined method section as BRICS System Design and Architecture, followed by two sections - Data Submission and Processing, Sharing and Access sections. We have integrated the Query Tool description within the section the Data Access section.

2. Under the data submission and processing section, we have added information on study level metadata, that is entered manually through a graphical user interface, when a BRICS instance is used. The examples of Metadata fields include title, organization, PI, data, funding source and ID’s, study type(s), and keywords that enable users to search for detailed information (e.g. clinical trial Grant ID(s), start and end dates for grants, therapeutic agents, sample size, publications, and forms used).

We have indicated that each of the BRICS instance exposes metadata and summary consistent with their respective program goals. We have provided an example, FITBIR provides a metadata visualization tool that graphically supports searching study identification (shown here https://fitbir.nih.gov/visualization).

3.Thank you for the suggestion. We have added information in the Data Sharing and Access section indicating that each instance of BRICS supports the data sharing policies consistent with their respective program. Research data is maintained in a private state until a year after the grant end date, and after that time, data is moved to a shared state where all users with approval from DAC can have access to the data. The DAC is comprised of government program officials responsible for each of the BRICS instances, who evaluate the data access requests and approve or disapprove the request. A detailed information for each of the BRICS instances can be gleaned from the site information (web site links) provided under the BRICS instance section.
